# Supplementary material for: A Likelihood Approach for Real-Time Calibration of Stochastic Compartmental Epidemic Models
Source: PLoS Comput Biol. 2017 Jan 17;13(1):e1005257. doi: 10.1371/journal.pcbi.1005257 (PMC5240920; doi:10.1371/journal.pcbi.1005257)
Supplement: S1 File — (TAR.GZ) [file pcbi.1005257.s014.tar.gz › HSPH_Online-SI-Revision/IPoi09/n10-extreme/n10-extreme_BENCH_table8.pdf]

1	R0	{2.68692, 12.5884}	{2.96307, 14.3219}	{{2.96307, 14.3219}, {2.96307, 14.3219}}	{10.2776, 13.7706}	inf	138.	173.645	{76., 321.}	52.4077	0.169274	newly	61.	70.6613	{32., 138.}	50.1058	0.178751	Reff	0.251496	0.195104	{0.178081, 0.21423}	22.4235	ar	9089.	9382.58	{9331., 9427.}	3.23007
2	R0	{1.79802, 12.5412}	{2.7947, 19.9815}	{{2.79269, 19.984}, {2.79269, 19.984}}	{55.4319, 59.3267}	inf	348.	718.367	{358., 924.}	108.18	0.259304	newly	172.	227.283	{114., 265.}	52.5484	0.159706	Reff	0.525561	0.303572	{0.23319, 0.312502}	49.8746	ar	7412.	9239.98	{9175., 9302.}	24.6625
3	R0	{2.6174, 10.1105}	{2.92375, 10.9639}	{{2.92375, 10.9639}, {2.92375, 10.9639}}	{11.7044, 8.44073}	inf	48.	81.3115	{35., 144.}	81.9672	0.213245	newly	29.	39.9344	{16., 70.}	66.7609	0.210991	Reff	0.238183	0.195273	{0.179226, 0.211095}	18.0157	ar	9109.	9353.07	{9308., 9392.}	2.67939
4	R0	{2.27532, 14.3149}	{2.93488, 18.8445}	{{2.93488, 18.8445}, {2.93488, 18.8445}}	{28.9876, 31.6426}	inf	238.	502.328	{234., 1074.}	114.52	0.265106	newly	95.	162.787	{73., 353.}	80.2243	0.202676	Reff	0.382254	0.231601	{0.198104, 0.286444}	39.4851	ar	8478.	9360.51	{9324., 9401.}	10.4094
5	R0	{2.96649, 18.0674}	{2.96947, 15.963}	{{2.96947, 15.963}, {2.96947, 15.963}}	{0.100455, 11.6475}	inf	369.	187.629	{82., 324.}	61.6706	0.417066	newly	132.	69.9194	{30., 104.}	61.0093	0.407547	Reff	0.177989	0.197168	{0.174902, 0.219147}	12.1991	ar	9476.	9383.39	{9320., 9450.}	0.982788
6	R0	{2.94778, 16.0002}	{2.95376, 15.278}	{{2.95376, 15.278}, {2.95376, 15.278}}	{0.202865, 4.51369}	inf	276.	390.565	{197., 722.}	56.7029	0.165139	newly	93.	151.887	{73., 267.}	72.6847	0.193566	Reff	0.21961	0.215296	{0.190813, 0.246344}	9.14557	ar	9321.	9372.5	{9323., 9414.}	0.589546
7	R0	{2.17562, 11.7184}	{2.99939, 13.7076}	{{2.99939, 13.7076}, {2.99939, 13.7076}}	{37.8637, 16.975}	inf	199.	166.274	{88., 232.}	48.6546	0.227053	newly	97.	70.3226	{35., 98.}	52.3113	0.275774	Reff	0.392917	0.188492	{0.172465, 0.203959}	52.0274	ar	8345.	9410.45	{9366., 9453.}	12.7675
8	R0	{2.7904, 17.8966}	{2.93488, 18.8445}	{{2.93488, 18.8445}, {2.93488, 18.8445}}	{5.17775, 5.29654}	inf	456.	502.328	{234., 1074.}	49.1156	0.183536	newly	172.	162.787	{73., 353.}	48.9325	0.213301	Reff	0.233277	0.231601	{0.198104, 0.286444}	13.0592	ar	9285.	9360.51	{9324., 9401.}	0.815346
9	R0	{2.51574, 16.376}	{2.96398, 17.7348}	{{2.96398, 17.7348}, {2.96398, 17.7348}}	{17.8174, 8.29751}	inf	368.	397.016	{193., 640.}	44.7099	0.170359	newly	131.	134.419	{63., 229.}	44.9643	0.181974	Reff	0.296857	0.215462	{0.186731, 0.244528}	28.6844	ar	8937.	9378.1	{9330., 9427.}	4.93562
10	R0	{2.56405, 18.1787}	{2.93488, 18.8445}	{{2.93488, 18.8445}, {2.93488, 18.8445}}	{14.4627, 3.66253}	inf	542.	502.328	{234., 1074.}	48.6056	0.217793	newly	185.	162.787	{73., 353.}	49.5436	0.231522	Reff	0.307686	0.231601	{0.198104, 0.286444}	27.4765	ar	9005.	9360.51	{9324., 9401.}	3.9479
11	R0	{2.475, 17.1709}	{2.42205, 19.4475}	{{2.42205, 19.4475}, {2.42205, 19.4475}}	{2.13939, 13.2585}	inf	441.	690.828	{292., 1241.}	76.9177	0.229875	newly	155.	229.	{90., 409.}	73.7709	0.231133	Reff	0.328185	0.416622	{0.311233, 0.470362}	29.7386	ar	8870.	8811.98	{8743., 8883.}	0.743887
12	R0	{2.14974, 11.8619}	{2.93488, 18.8445}	{{2.93488, 18.8445}, {2.93488, 18.8445}}	{36.5226, 58.8658}	inf	247.	852.525	{430., 1695.}	245.152	0.477411	newly	113.	279.393	{144., 572.}	147.512	0.3312	Reff	0.367391	0.274344	{0.216007, 0.396796}	34.1931	ar	8414.	9360.51	{9324., 9401.}	11.2492
13	R0	{2.20385, 14.4767}	{2.64373, 19.7855}	{{2.64373, 19.7855}, {2.64373, 19.7855}}	{19.9596, 36.6713}	inf	257.	744.254	{365., 1308.}	189.593	0.402276	newly	114.	234.508	{111., 420.}	108.187	0.261652	Reff	0.397134	0.325875	{0.26781, 0.402111}	24.3546	ar	8347.	9088.49	{9032., 9142.}	8.88334
14	R0	{2.35671, 13.8357}	{2.96947, 15.963}	{{2.96947, 15.963}, {2.96947, 15.963}}	{26.0007, 15.3754}	inf	237.	265.145	{123., 459.}	53.008	0.190373	newly	104.	98.3548	{46., 170.}	54.3114	0.233393	Reff	0.328997	0.203356	{0.17698, 0.225086}	38.189	ar	8719.	9383.39	{9320., 9450.}	7.61999
15	R0	{1.91368, 10.1082}	{2.86656, 18.9813}	{{2.86656, 18.9813}, {2.86656, 18.9813}}	{49.7931, 87.7812}	inf	135.	443.197	{233., 950.}	228.294	0.440885	newly	76.	145.049	{74., 324.}	94.1329	0.220356	Reff	0.503681	0.237055	{0.205246, 0.268597}	52.9354	ar	7486.	9306.52	{9263., 9354.}	24.3191
16	R0	{2.48727, 14.882}	{2.86118, 15.5722}	{{2.86118, 15.5722}, {2.86118, 15.5722}}	{15.0329, 4.63782}	inf	327.	387.508	{199., 708.}	44.6333	0.151624	newly	131.	148.803	{72., 262.}	44.5501	0.162425	Reff	0.299467	0.229954	{0.204002, 0.254359}	24.033	ar	8894.	9309.66	{9255., 9350.}	4.67344
17	R0	{2.46795, 13.1915}	{2.99939, 13.7076}	{{2.99939, 13.7076}, {2.99939, 13.7076}}	{21.5337, 3.91237}	inf	235.	248.839	{132., 347.}	44.4887	0.163122	newly	107.	103.968	{53., 152.}	45.6437	0.185734	Reff	0.326757	0.194912	{0.175764, 0.210557}	40.6858	ar	8796.	9410.45	{9366., 9453.}	6.98558
18	R0	{2.96603, 16.7543}	{2.96947, 15.963}	{{2.96947, 15.963}, {2.96947, 15.963}}	{0.11598, 4.72297}	inf	304.	371.548	{174., 625.}	53.7882	0.175181	newly	107.	137.371	{64., 227.}	57.5671	0.181579	Reff	0.209995	0.212552	{0.182919, 0.237855}	10.3631	ar	9394.	9383.39	{9320., 9450.}	0.407261
19	R0	{2.25216, 9.90465}	{2.99939, 13.7076}	{{2.99939, 13.7076}, {2.99939, 13.7076}}	{33.1784, 38.3956}	inf	114.	248.839	{132., 347.}	119.015	0.269887	newly	62.	103.968	{53., 152.}	74.6098	0.18292	Reff	0.321383	0.194912	{0.175764, 0.210557}	39.7481	ar	8631.	9410.45	{9366., 9453.}	9.03084
20	R0	{2.70837, 18.1923}	{2.93488, 18.8445}	{{2.93488, 18.8445}, {2.93488, 18.8445}}	{8.36333, 3.58503}	inf	470.	852.525	{430., 1695.}	85.9016	0.219844	newly	154.	279.393	{144., 572.}	85.7462	0.216806	Reff	0.256212	0.274344	{0.216007, 0.396796}	20.2091	ar	9183.	9360.51	{9324., 9401.}	1.93301
21	R0	{2.30288, 15.5877}	{2.79269, 19.984}	{{2.79269, 19.984}, {2.79269, 19.984}}	{21.2695, 28.2036}	inf	338.	586.3	{271., 980.}	81.9625	0.201799	newly	137.	184.883	{80., 297.}	60.3528	0.177589	Reff	0.344741	0.280242	{0.224253, 0.290998}	30.7765	ar	8674.	9238.12	{9175., 9301.}	6.50354
22	R0	{2.82208, 10.7544}	{2.91946, 11.8546}	{{2.91946, 11.8546}, {2.91946, 11.8546}}	{3.45065, 10.2302}	inf	91.	146.517	{71., 237.}	71.4103	0.195932	newly	39.	68.7167	{31., 106.}	83.8889	0.218384	Reff	0.196981	0.200752	{0.183342, 0.222755}	6.11538	ar	9333.	9349.67	{9293., 9392.}	0.3943
23	R0	{1.69923, 10.1366}	{2.93488, 18.8445}	{{2.93488, 18.8445}, {2.93488, 18.8445}}	{72.7182, 85.9055}	inf	136.	502.328	{234., 1074.}	269.359	0.491136	newly	84.	162.787	{73., 353.}	98.79	0.235642	Reff	0.525912	0.231601	{0.198104, 0.286444}	55.9621	ar	7055.	9360.51	{9324., 9401.}	32.6791
24	R0	{2.64944, 19.054}	{2.85956, 19.8999}	{{2.85956, 19.8999}, {2.85956, 19.8999}}	{7.93073, 4.43949}	inf	411.	457.984	{236., 739.}	49.292	0.182369	newly	150.	143.689	{73., 249.}	46.9617	0.203722	Reff	0.26097	0.24063	{0.207318, 0.275662}	15.613	ar	9147.	9302.69	{9253., 9345.}	1.70207
25	R0	{2.53191, 10.9214}	{2.8833, 17.1847}	{{2.8833, 17.1847}, {2.8833, 17.1847}}	{13.8785, 57.3489}	inf	104.	522.951	{248., 947.}	402.837	0.634313	newly	53.	183.115	{87., 326.}	245.5	0.47103	Reff	0.259774	0.243473	{0.211058, 0.270742}	14.2677	ar	9009.	9313.56	{9263., 9361.}	3.38059
26	R0	{1.89155, 6.76944}	{2.9884, 12.8326}	{{2.9884, 12.8326}, {2.9884, 12.8326}}	{57.9868, 89.5666}	inf	18.	147.548	{72., 260.}	719.713	0.848142	newly	10.	63.2258	{31., 112.}	532.258	0.733614	Reff	0.5058	0.188086	{0.172431, 0.204407}	62.8142	ar	7348.	9404.84	{9361., 9451.}	27.9918
27	R0	{2.63149, 15.6979}	{2.7499, 15.3124}	{{2.7499, 15.3124}, {2.7499, 15.3124}}	{4.49973, 2.45574}	inf	374.	462.242	{217., 769.}	51.4188	0.176766	newly	136.	178.532	{84., 301.}	55.2538	0.178826	Reff	0.26236	0.260243	{0.227417, 0.292589}	9.2538	ar	9104.	9203.37	{9154., 9249.}	1.09505
28	R0	{2.85141, 12.6992}	{2.91946, 11.8546}	{{2.91946, 11.8546}, {2.91946, 11.8546}}	{2.38654, 6.65081}	inf	136.	92.3333	{42., 155.}	49.3873	0.292809	newly	59.	43.3833	{18., 78.}	50.0847	0.28628	Reff	0.201595	0.196509	{0.180715, 0.212245}	5.79603	ar	9317.	9349.67	{9293., 9392.}	0.472613
29	R0	{2.54244, 12.9085}	{2.93488, 18.8445}	{{2.93488, 18.8445}, {2.93488, 18.8445}}	{15.4356, 45.9852}	inf	177.	659.951	{313., 1339.}	272.854	0.501432	newly	83.	216.443	{101., 425.}	160.774	0.341979	Reff	0.256278	0.248863	{0.205735, 0.333402}	17.469	ar	9034.	9360.51	{9324., 9401.}	3.61422
30	R0	{2.10982, 11.7636}	{2.93488, 18.8445}	{{2.93488, 18.8445}, {2.93488, 18.8445}}	{39.1057, 60.1933}	inf	182.	502.328	{234., 1074.}	176.401	0.366438	newly	78.	162.787	{73., 353.}	111.854	0.25852	Reff	0.400444	0.231601	{0.198104, 0.286444}	42.164	ar	8224.	9360.51	{9324., 9401.}	13.8194
31	R0	{2.76312, 15.5396}	{2.95376, 15.278}	{{2.95376, 15.278}, {2.95376, 15.278}}	{6.89945, 1.68344}	inf	308.	390.565	{197., 722.}	49.1674	0.157443	newly	111.	151.887	{73., 267.}	54.7079	0.163848	Reff	0.234589	0.215296	{0.190813, 0.246344}	11.9584	ar	9220.	9372.5	{9323., 9414.}	1.65401
32	R0	{2.21727, 15.5439}	{2.42205, 19.4475}	{{2.42205, 19.4475}, {2.42205, 19.4475}}	{9.23568, 25.1134}	inf	307.	690.828	{292., 1241.}	129.619	0.300752	newly	111.	229.	{90., 409.}	116.278	0.280845	Reff	0.413964	0.416622	{0.311233, 0.470362}	26.7483	ar	8359.	8811.98	{8743., 8883.}	5.4191
33	R0	{2.72912, 10.8159}	{2.99939, 13.7076}	{{2.99939, 13.7076}, {2.99939, 13.7076}}	{9.90319, 26.7356}	inf	49.	166.274	{88., 232.}	239.335	0.453313	newly	25.	70.3226	{35., 98.}	181.29	0.36837	Reff	0.222969	0.188492	{0.172465, 0.203959}	16.4416	ar	9196.	9410.45	{9366., 9453.}	2.33201
34	R0	{2.20928, 10.1308}	{2.92375, 10.9639}	{{2.92375, 10.9639}, {2.92375, 10.9639}}	{32.3395, 8.22344}	inf	59.	49.5738	{22., 91.}	49.2081	0.251897	newly	37.	24.0492	{9., 39.}	51.9273	0.327647	Reff	0.360775	0.192876	{0.178934, 0.207879}	46.5384	ar	8388.	9353.07	{9308., 9392.}	11.5053
35	R0	{2.10103, 15.2673}	{2.93488, 18.8445}	{{2.93488, 18.8445}, {2.93488, 18.8445}}	{39.6877, 23.4305}	inf	355.	381.803	{179., 807.}	50.2193	0.192675	newly	143.	125.344	{59., 259.}	51.8056	0.244163	Reff	0.409701	0.219197	{0.192235, 0.261204}	46.4983	ar	8264.	9360.51	{9324., 9401.}	13.2685
36	R0	{2.74914, 19.2775}	{2.64373, 19.7855}	{{2.64373, 19.7855}, {2.64373, 19.7855}}	{3.83429, 2.6352}	inf	508.	744.254	{365., 1308.}	60.5737	0.1782	newly	161.	234.508	{111., 420.}	61.6484	0.183315	Reff	0.249622	0.325875	{0.26781, 0.402111}	30.5476	ar	9301.	9088.49	{9032., 9142.}	2.28479
37	R0	{2.78161, 16.0136}	{2.96398, 17.7348}	{{2.96398, 17.7348}, {2.96398, 17.7348}}	{6.55627, 10.7484}	inf	295.	536.323	{269., 894.}	86.6156	0.219191	newly	117.	183.516	{87., 288.}	67.9901	0.184743	Reff	0.232264	0.228566	{0.190584, 0.272983}	12.5497	ar	9240.	9378.1	{9330., 9427.}	1.49455
38	R0	{2.4454, 8.33501}	{2.9884, 12.8326}	{{2.9884, 12.8326}, {2.9884, 12.8326}}	{22.205, 53.9602}	inf	27.	147.548	{72., 260.}	446.476	0.672051	newly	18.	63.2258	{31., 112.}	251.254	0.478342	Reff	0.282933	0.188086	{0.172431, 0.204407}	33.5227	ar	8853.	9404.84	{9361., 9451.}	6.23335
39	R0	{2.9154, 12.3861}	{2.9884, 12.8326}	{{2.9884, 12.8326}, {2.9884, 12.8326}}	{2.50394, 3.60485}	inf	88.	147.548	{72., 260.}	74.9267	0.194848	newly	42.	63.2258	{31., 112.}	63.3641	0.177094	Reff	0.190376	0.188086	{0.172431, 0.204407}	5.49606	ar	9371.	9404.84	{9361., 9451.}	0.417555
40	R0	{2.40923, 15.1963}	{2.96398, 17.7348}	{{2.96398, 17.7348}, {2.96398, 17.7348}}	{23.026, 16.7047}	inf	294.	536.323	{269., 894.}	87.1407	0.22019	newly	115.	183.516	{87., 288.}	69.8738	0.187764	Reff	0.339701	0.228566	{0.190584, 0.272983}	34.5924	ar	8735.	9378.1	{9330., 9427.}	7.3623
41	R0	{2.63137, 15.238}	{2.93488, 18.8445}	{{2.93488, 18.8445}, {2.93488, 18.8445}}	{11.5343, 23.6678}	inf	281.	502.328	{234., 1074.}	86.2085	0.214175	newly	89.	162.787	{73., 353.}	89.5745	0.219249	Reff	0.24998	0.231601	{0.198104, 0.286444}	16.0989	ar	9150.	9360.51	{9324., 9401.}	2.30064
42	R0	{2.48769, 19.4593}	{2.42205, 19.4475}	{{2.42205, 19.4475}, {2.42205, 19.4475}}	{2.63859, 0.0606394}	inf	467.	690.828	{292., 1241.}	71.2914	0.22387	newly	165.	229.	{90., 409.}	68.3386	0.226814	Reff	0.312454	0.416622	{0.311233, 0.470362}	34.2643	ar	8932.	8811.98	{8743., 8883.}	1.34561
43	R0	{2.61199, 10.214}	{2.99499, 12.6493}	{{2.99519, 12.6436}, {2.99519, 12.6436}}	{14.663, 23.8425}	inf	68.	218.339	{89., 332.}	221.086	0.420366	newly	40.	98.0806	{39., 145.}	148.589	0.311437	Reff	0.251796	0.193451	{0.17432, 0.213258}	23.8249	ar	9061.	9405.27	{9353., 9456.}	3.79952
44	R0	{2.50994, 18.3382}	{2.46477, 18.9032}	{{2.46477, 18.9032}, {2.46477, 18.9032}}	{1.79964, 3.081}	inf	483.	658.1	{272., 1078.}	57.0945	0.18498	newly	173.	221.633	{91., 370.}	55.5106	0.189855	Reff	0.350137	0.387088	{0.294294, 0.453518}	23.0415	ar	8827.	8883.57	{8780., 8947.}	0.817945
45	R0	{2.44283, 16.8338}	{2.93488, 18.8445}	{{2.93488, 18.8445}, {2.93488, 18.8445}}	{20.1426, 11.9444}	inf	399.	659.951	{313., 1339.}	74.621	0.195511	newly	142.	216.443	{101., 425.}	66.1856	0.180092	Reff	0.308285	0.248863	{0.205735, 0.333402}	26.7133	ar	8878.	9360.51	{9324., 9401.}	5.43487
46	R0	{2.51815, 18.1462}	{2.64373, 19.7855}	{{2.64373, 19.7855}, {2.64373, 19.7855}}	{4.98699, 9.03385}	inf	537.	282.286	{127., 503.}	56.6847	0.386258	newly	173.	87.7143	{40., 167.}	57.464	0.401203	Reff	0.31905	0.267739	{0.242959, 0.297155}	16.9262	ar	8978.	9088.49	{9032., 9142.}	1.23918
47	R0	{2.88885, 16.8987}	{2.93488, 18.8445}	{{2.93488, 18.8445}, {2.93488, 18.8445}}	{1.59337, 11.5145}	inf	414.	659.951	{313., 1339.}	69.9335	0.186732	newly	129.	216.443	{101., 425.}	77.2906	0.198484	Reff	0.22533	0.248863	{0.205735, 0.333402}	15.9888	ar	9337.	9360.51	{9324., 9401.}	0.352555
48	R0	{2.58709, 13.8786}	{2.95376, 15.278}	{{2.95376, 15.278}, {2.95376, 15.278}}	{14.1731, 10.0831}	inf	256.	390.565	{197., 722.}	64.1318	0.177714	newly	109.	151.887	{73., 267.}	56.1261	0.165631	Reff	0.243704	0.215296	{0.190813, 0.246344}	14.3528	ar	9146.	9372.5	{9323., 9414.}	2.47649
49	R0	{2.42959, 10.7132}	{2.99939, 13.7076}	{{2.99939, 13.7076}, {2.99939, 13.7076}}	{23.4525, 27.9506}	inf	116.	248.839	{132., 347.}	115.462	0.263309	newly	60.	103.968	{53., 152.}	78.871	0.190501	Reff	0.288149	0.194912	{0.175764, 0.210557}	33.1709	ar	8863.	9410.45	{9366., 9453.}	6.17682
50	R0	{2.60513, 13.2796}	{2.96398, 17.7348}	{{2.96398, 17.7348}, {2.96398, 17.7348}}	{13.7747, 33.5492}	inf	243.	714.5	{362., 1216.}	194.033	0.410522	newly	108.	244.21	{125., 432.}	126.389	0.295022	Reff	0.256605	0.248138	{0.201847, 0.303512}	16.7576	ar	9095.	9378.1	{9330., 9427.}	3.11266
